# Characterization of Structure and Dynamics of the Guanidine‐II Riboswitch from *Escherichia coli* by NMR Spectroscopy and Small‐Angle X‐ray Scattering (SAXS)

**DOI:** 10.1002/cbic.202100564

**Published:** 2021-12-09

**Authors:** Tatjana Schamber, Oliver Binas, Andreas Schlundt, Anna Wacker, Harald Schwalbe

**Affiliations:** ^1^ Institute for Organic Chemistry and Chemical Biology Johann Wolfgang Goethe University Max-von-Laue-Str. 7 60438 Frankfurt/Main Germany; ^2^ Institute for Molecular Biosciences Johann Wolfgang Goethe University Max-von-Laue-Str. 9 60438 Frankfurt/Main Germany; ^3^ Center for Biomolecular Magnetic Resonance Institute for Organic Chemistry and Chemical Biology Johann Wolfgang Goethe University Max-von-Laue-Str. 7–9 60438 Frankfurt/Main Germany

**Keywords:** aptamers, guanidine riboswitch, NMR spectroscopy, RNA, structural biology

## Abstract

Riboswitches are regulatory RNA elements that undergo functionally important allosteric conformational switching upon binding of specific ligands. The here investigated guanidine‐II riboswitch binds the small cation, guanidinium, and forms a kissing loop‐loop interaction between its P1 and P2 hairpins. We investigated the structural changes to support previous studies regarding the binding mechanism. Using NMR spectroscopy, we confirmed the structure as observed in crystal structures and we characterized the kissing loop interaction upon addition of Mg^2+^ and ligand for the riboswitch aptamer from *Escherichia coli*. We further investigated closely related mutant constructs providing further insight into functional differences between the two (different) hairpins P1 and P2. Formation of intermolecular interactions were probed by small‐angle X‐ray scattering (SAXS) and NMR DOSY data. All data are consistent and show the formation of oligomeric states of the riboswitch induced by Mg^2+^ and ligand binding.

## Graphical abstract

**Gdn‐II riboswitch aptamer** from *E. coli* with wild‐type and loop‐mutated constructs were investigated in the context of ligand interactions. Based on NMR spectroscopy and small‐angle X‐ray scattering (SAXS) data, previous crystal structures were confirmed, and an intermolecular ligand binding mechanism with the P1 loop as interaction interface instead of the postulated (intramolecular) P1‐P2 kissing loop was found.

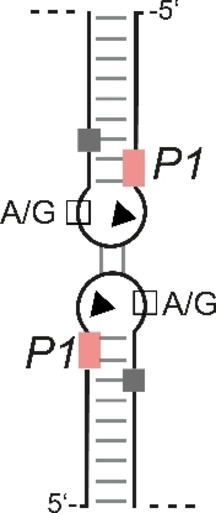

## Introduction

In bacteria, riboswitches are wide‐spread regulatory elements primarily located in the 5’‐untranslated region (5’‐UTR) of mRNAs. They control gene expression mainly at the level of translation or transcription in response to binding of molecules of low molecular weight. While a large variety of riboswitches are known to sense various cell‐metabolites^[1]^ including TPP,[^2^, ^3^] FMN,^[4]^ preQ_1_,^[5]^ amino acids,^[6]^ or purines,^[7]^ as well as the secondary messengers 3’,3’‐cGAMP,^[8]^ c‐di‐GMP,^[9]^ ZMP^[10]^ and even small ions (F^−^, Mg^2+^),[^11^, ^12^] riboswitches sensing the cytotoxic guanidinium cation were only recently discovered.^[13]^ Guanidinium (Gdm^+^) is the protonated form of guanidine, possesses D_3h_ symmetry and is protonated under physiological conditions due to its high pK_A_ value of 13.6. It is able to provide six hydrogen bonds for binding to hydrogen acceptor sites in RNA.^[14]^ The Gdm^+^ cation is significantly larger than the ubiquitous RNA‐binding cation Mg^2+^, also larger than F^−^.^[11]^

The putative biological function of free guanidine has been discussed for long but was not ascertained due to the absence of biological receptors. In fact, new insight came from the discovery of riboswitches that function as Gdm^+^‐binding receptors.[^15^, ^16^] These guanidine riboswitches (Gdn) sense Gdm^+^ with high sensitivity and specificity.^[17]^ Derived from the genomic location of the Gdm^+^‐sensing riboswitches, Gdm^+^ has then been proposed to play an important role in the detoxification of cells via its carboxylation^[13]^ but also to serve as a source of nitrogen.^[18]^

To date, four different classes of Gdm^+^‐sensing riboswitches have been discovered termed guanidine‐I to guanidine‐IV.[^15^, ^18^, ^19^] Many of these Gdm^+^‐sensing riboswitches are biologically well characterized and structural models of isolated hairpins were reported for all the four riboswitch classes including Gdn‐II from *Escherichia coli* (PDB: 5NDI), from *Gloeobacter violaceus* (5NOM and 5NDH) and from *Pseudomonas aeruginosa* (5VJ9).[^14^, ^20^] The structures reveal striking similarities of ligand binding pockets between these different riboswitches.^[21]^

**Figure 1:**
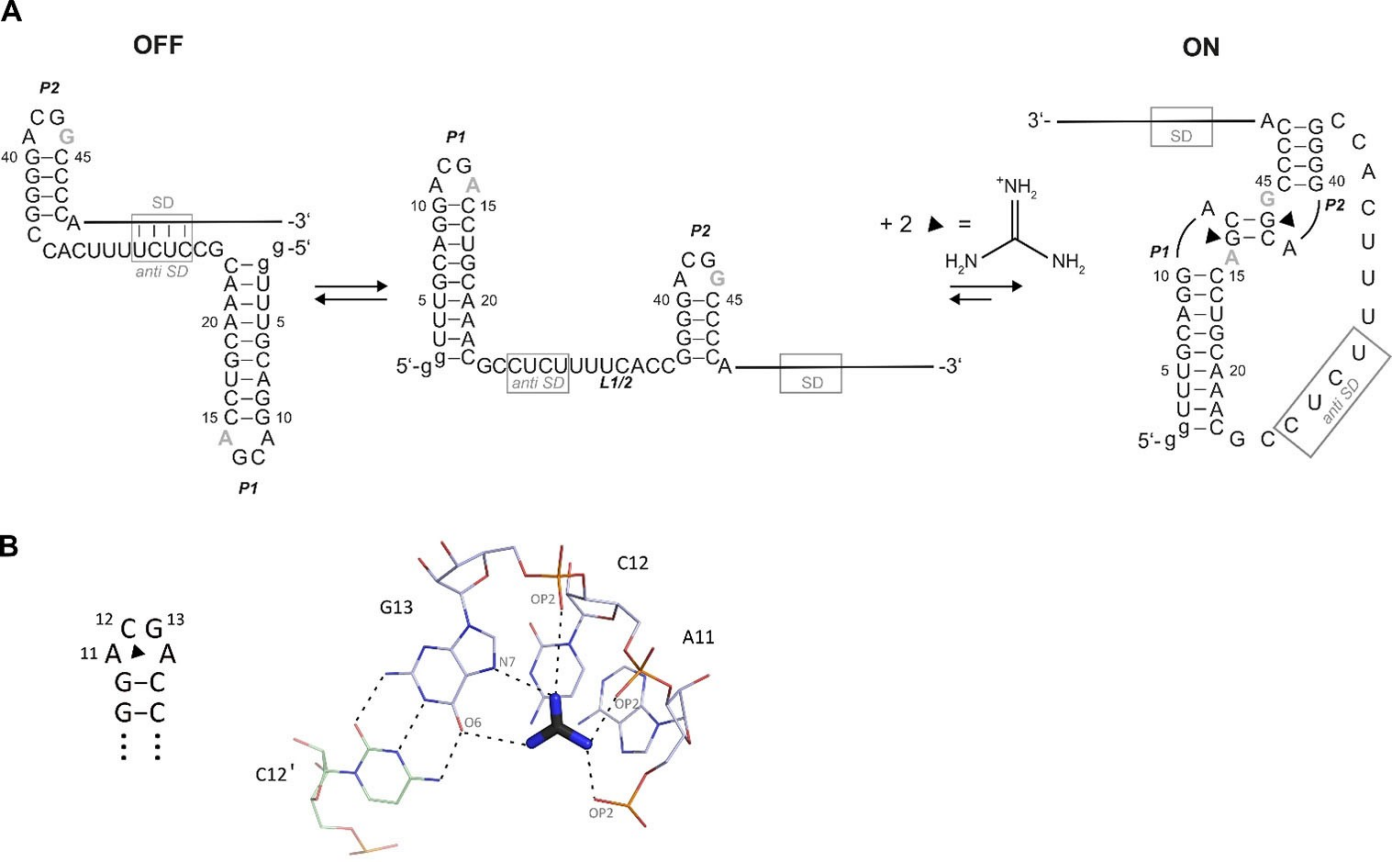
(A) Secondary structures of the investigated aptamer within the Gdn‐II riboswitch and the proposed mechanism of switching.^[20]^ The structural elements P1, P2 and linker L1/2 are annotated accordingly. ‘SD’ and ‘*anti*‐SD’ denote the Shine‐Dalgarno and complementary *anti*‐Shine‐Dalgarno sequences, respectively. The current model of the mechanism of switching of the full‐length Gdn‐II riboswitch including the expression platform is shown. (B) Representation of the ligand binding pocket of P1 depicting hydrogen bonds for Gdm^+^ to the Hoogsteen side of guanosine (via O6 and N7) and the backbone phosphates (right). The corresponding sequence is shown on the left.

For a Gdn‐II class riboswitch aptamer domain, it was shown that binding of Gdm^+^ to the Gdm^+^‐sensing riboswitches induces kissing loop‐loop interactions leading to the formation of two GC base pairs between its two hairpin elements (Figure 1A). The Gdm^+^ ligand binds via hydrogen bonds to the Hoogsteen side of the third loop nucleobase guanosine G13 (O6, N7) and the backbone phosphodiesters. In addition, π‐ and ionic interactions stabilize ligand binding (Figure 1B).^[14]^ Up to now, only hairpins with *ACG**A*** loop motifs have been investigated at the structural level but not the equally frequent *ACG**G*** loop motifs.^[22]^ Likewise, the possibility of hetero dimerization involving two independent and different looped hairpins has escaped detailed (structural) analysis. We here investigate the Gdn‐II class riboswitch aptamer from *E. coli* (Figure 1A) that is part of the *sugE* gene which codes for a multi drug efflux pump^[17]^ by NMR spectroscopy and small‐angle X‐ray scattering (SAXS). The riboswitch consists of the mini‐*ykkC* motif^[22]^ that contains two linked GC rich stem‐loop domains P1 and P2. Both stems are capped by *ACG**R*** loop motifs.^[14]^ The kissing loop‐loop interaction was proposed to up‐regulate *SugE* through weakening of the interaction of linker L1/2 with a putative downstream Shine‐Dalgarno sequence (Figure 1A). The increased accessibility to the Shine‐Dalgarno ribosome binding sites is assumed to promote translation initiation.^[17]^ Kissing loop‐loop interactions upon ligand binding have previously been reported in various riboswitches but striking differences are observed.^[23]^ In guanine‐ and adenine‐sensing riboswitches, the ligand binding site is remote from the kissing loop‐loop interaction site. Formation of the kissing loop‐loop interaction leads to an allosteric stabilization of the RNA‐ligand interaction. By contrast, in the Gdn‐II riboswitch, the ligand binding site is located in close proximity to the kissing loop‐loop interaction site. While the interaction between P1 and P2 in Gdn‐II has been studied systematically on a mimic of the double stem‐loop RNA,^[24]^ its existence has not been shown for the wild‐type sequence, i. e. including the linker L1/2 and two different *ACGR* loops. In this study, we provide evidence that the two GC base pairs form the putative kissing loop‐loop interaction and map binding sites for the ligand to various relevant constructs of the riboswitch at atomic resolution. We further investigate mutant constructs to delineate functional differences between the two hairpin domains.

## Results and Discussion

### NMR resonance assignment

**Figure 2:**
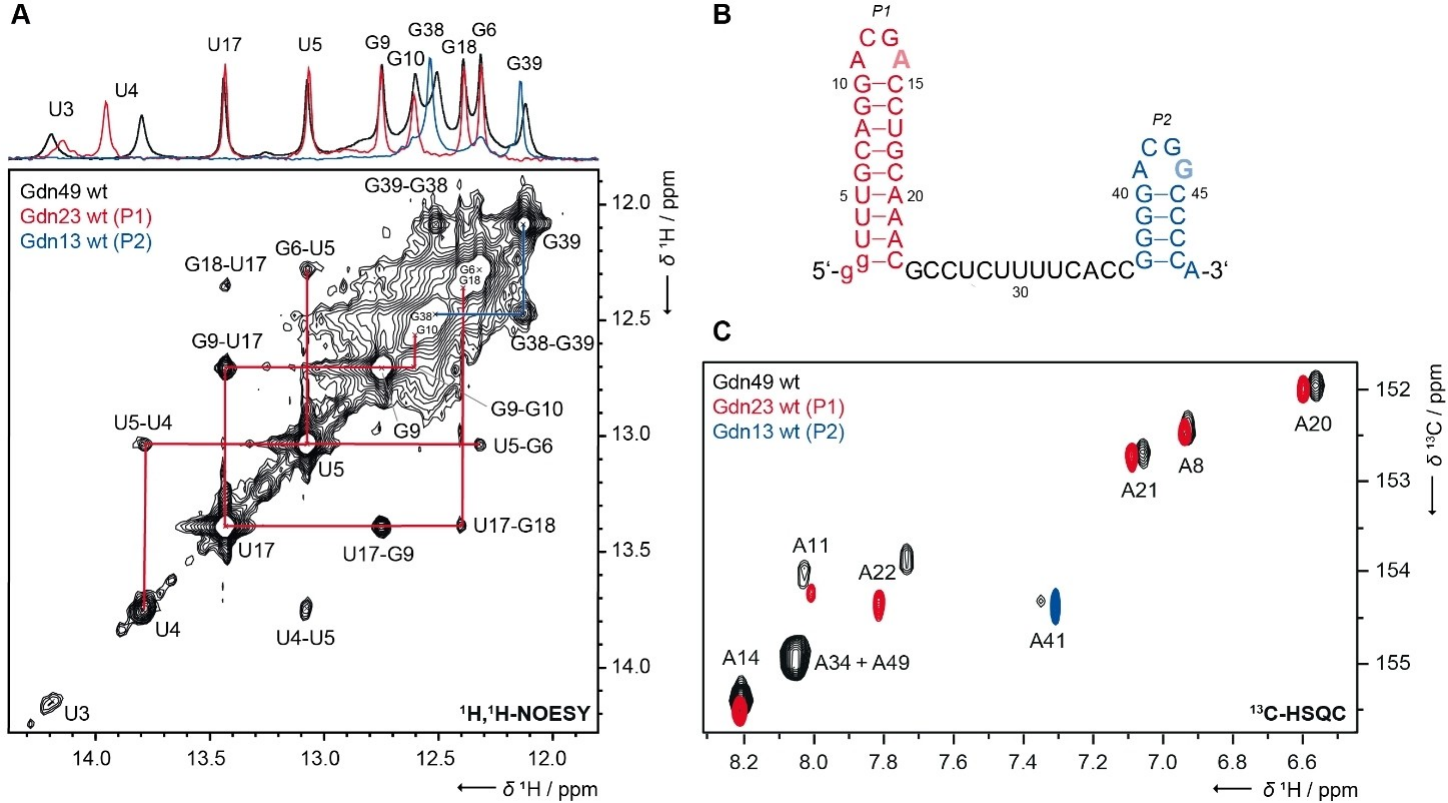
NMR resonance assignments. (A) Imino region of ^1^H,^1^H‐NOESY spectra of Gdn49 wt annotated with assignments. Color‐coding indicates resonances from either P1 (red) or P2 (blue). Data were measured at 600 MHz, 2048×768 points, 160 scans and 150 ms mixing time. The sample contained 600 μM RNA. (B) Secondary structure of the investigated Gdn‐II riboswitch from *E. coli*. Full‐length aptamer is Gdn49 wt, P1 is Gdn23 wt with *ACG**A*** loop motif in red and P2 is Gdn13 wt with *ACG**G*** loop motif in blue. (C) ^13^C‐HSQC spectra of adenosines C2‐H2 recorded from Gdn49 wt and the structural elements P1 (Gdn23 wt) and P2 (Gdn13 wt). Data were measured at 950/238 MHz, 2048×128 points, 256 scans with 720 μM of Gdn13 wt, 800/201 MHz, 1024×128 points, 512 scans with 660 μM of Gdn23 wt and 950/238 MHz, 1332×128 points, 2 scans with 800 μM ^13^C,^15^N‐labeled Gdn49 wt.

Following a divide‐and‐conquer‐strategy,^[25]^ we conducted NMR experiments of the wild‐type (wt) Gdn‐II aptamer domain from *E. coli* containing 49 nucleotides (nts) RNA (Gdn49 wt) and assigned its NMR resonances. We used two constructs consisting of the first stem‐loop with 23 nts (Gdn23 wt as P1) and the second stem‐loop with 13 nts (Gdn13 wt as P2). Both stem‐loops are present in the full‐length aptamer Gdn49 wt (Figure 2B) and we could in fact observe comparable sets of imino signals from nucleobases involved in base pairing in ^1^H‐1D as well as 2D‐^1^H,^1^H‐NOESY experiments (Figure 2A). Signals at 12.1 ppm (assigned as G39) and 12.5 ppm (assigned as G38) are present in Gdn13 wt (blue) and Gdn49 wt (black) but not in Gdn23 wt (red), and signals of Gdn23 wt are present in Gdn49 wt but not in Gdn13 wt, a finding that is also supported from analysis of ^1^H,^13^C‐correlations, e. g. C2‐H2 (Figure 2C and Supporting Figure S1).

**Figure 3:**
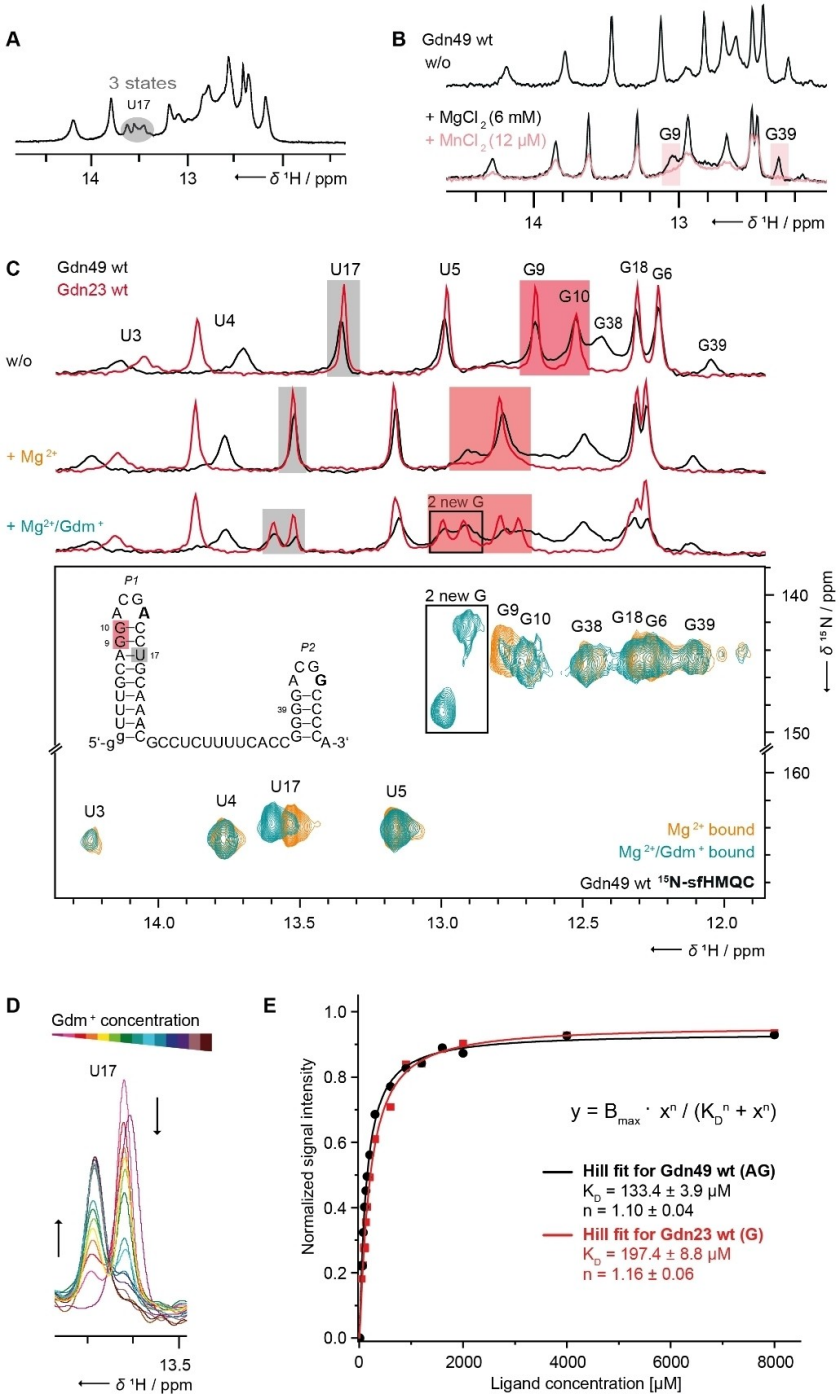
Mg^2+^ and ligand dependent structural changes observed by CSP. (A) Three states detected for U17 in sub‐stoichiometric titration experiments (unbound, Mg^2+^‐ and Mg^2+^/Gdm^+^‐bound) in ^1^H‐1D NMR spectra of Gdn49 wt in presence of 3 mM Mg^2+^ and 430 μM Gdm^+^. Data were measured at 600 MHz, room temperature, 8192 points, 512 scans with 215 μM of Gdn49 wt. (B) Determination of Mg^2+^ binding site through Mn^2+^‐induced paramagnetic relaxation enhancement for Gdn49 wt with ^1^H‐1D NMR spectra of Gdn49 wt unbound, in presence of 6 mM Mg^2+^ and in addition of 12 μM Mn^2+^ (0.2 %). Data were measured at 600 MHz, room temperature, 4096 points, 1024 scans with 20 μM of RNA. (C) *1D spectra*: Ligand titration states for Gdn23 wt and Gdn49 wt. Imino CSPs marked in gray for U17 and in red for G9, G10 and two new arising signals, respectively. ^1^H‐1D NMR spectra unbound, in presence of 5 mM Mg^2+^ and in addition of 320 μM Gdm^+^. Data were measured at 600 MHz, room temperature, 4096 points, 1024 scans with 20 μM RNA. *2D spectra*: Verifying of ligand‐induced GC base pairs in ^15^N‐SOFAST‐HMQC spectra of Gdn49 wt before (orange) and after (cyan) addition of Gdm^+^ in presence of 5 mM Mg^2+^. Data were measured at 600 MHz, room temperature, 36 points and 64 scans. Sample contained 100 μM RNA of Gdn49 wt. (D) Gdm^+^ dependent decrease and increase of signal intensity in ^1^H‐1D NMR spectra for the K_D_ determination. The signal of U17 in Gdn49 wt was analyzed. The color coding corresponds to a rainbow gradient from low concentration of Gdm^+^ in red to high concentration in violet. ^1^H‐1D NMR spectra in presence of 5 mM Mg^2+^ and in addition of up to 8000 μM Gdm^+^. Data were measured at 700 MHz, room temperature, 2048 points, 768 scans with 50 μM of RNA. (E) Plot of K_D_‐determination via ^1^H‐1D NMR titration experiments (Supporting Figure S5) for imino signal U17 of Gdn23 wt (red) and Gdn49 wt (black). The resulted values were determined by the Hill equation fit.

^1^H chemical shift assignment was obtained from analysis of 2D‐^1^H,^1^H‐NOESY experiments. Sequentially connected Watson‐Crick base pairs AU and GC were identified in the imino‐imino cross peak region of the NOESY spectra. The assignment was confirmed by ^1^H,^15^N‐correlation spectra (as depicted for the bound state in Figure 3C). As a result, the sequential assignment for all guanosine and uridine residues in the RNA stems was obtained.^[25]^ Note that the minor chemical shift differences of the U3 and U4 resonances for the Gdn23 wt and Gdn49 wt are typically observed in divide‐and‐conquer approaches and are likely due to the different stem stability in presence or absence of the linker. The shift of U4 is larger than of U3 but the reason for this remains unclear. We did not assign the imino proton of G40 which partially overlaps with G6 or G10. Further, imino protons of the terminal helical base pairs in P1 and P2 were not observed due to solvent exchange. We also observed weak NMR signals at 10.3 ppm for the constructs including P2 (Gdn13 wt and Gdn49 wt, Supporting Figure S2), indicating the presence of a non‐canonical base pair potentially stabilized by a G−G Hoogsteen interaction.

### Ligand interaction in guanidine‐II wild‐type constructs of *E. coli*

In NMR titration experiments, we characterized the interaction of the Gdn‐II riboswitch with Mg^2+^ and the ligand Gdm^+^, respectively, by monitoring chemical shift perturbations (CSPs) upon increasing concentrations of both ligands. It was previously established that Gdm^+^ binding requires the presence of Mg^2+^.^[26]^ We were able to detect the simultaneous population of three different long‐lived conformational states (unbound, Mg^2+^‐bound and Mg^2+^/Gdm^+^‐bound) using the imino proton signal of U17 as a reporter signal (Figure 3A). We deliberately started by adding only 3 mM Mg^2+^ which does not suffice to saturate the Mg^2+^ binding capacity of the riboswitch at an RNA concentration of 215 μM for Gdn49 wt. Under this condition, we observed signal changes compared to the unbound state both, for the Mg^2+^ bound state and for the Mg^2+^/Gdm^+^‐bound state after adding 430 μM Gdm^+^. Specific Mg^2+^ binding sites involving nucleotides G9 (P1) and G39 (P2) could be identified via Mn^2+^‐induced paramagnetic relaxation enhancement (PRE)^[27]^ (Figure 3B). These Mn^2+^‐PRE experiments typically involve quantification of the change in signal intensity when adding sub‐stoichiometric amounts of Mn^2+^ competing with Mg^2+^ for binding under fast exchange conditions.^[27]^ The identified nucleotides G9 (P1) and G39 (P2) are located in close proximity to the ligand binding site adjacent to the loop, and consequently the corresponding signals vanished upon addition of MnCl_2_. For the remaining signals, a significantly smaller decrease intensity and signal broadening was observed. To define this decrease, we used the integral ratio between spectra before and after adding of MnCl_2_ (see percentages of integrals for remaining signals in Figure 3B).

We further characterized Gdm^+^‐binding in the presence of 5 mM Mg^2+^. Imino resonances of U17 (marked in gray, Figure 3C *1D* spectra and Supporting Figure S3), G9 and G10 (marked in red) of P1 showed significant CSPs. We noted that U17 shows larger changes than G9 and G10 but we cannot explain this. Upon addition of Gdm^+^ (up to 8 mM), the formation of two GC kissing base pairs is reasoned by the detection of two new imino proton signals (Figure 3C *2D spectra*), in line with the base pairs observed in crystal structures.^[19]^ For Gdn23 wt and Gdn49 wt, we see shifts both, for Mg^2+^ and additional shift of Gdm^+^ interaction. By contrast, in P2, we only observed Mg^2+^‐induced CSPs, but no further shifts in effect of Gdm^+^ as reporter signals indicating no interaction of Gdm^+^ with the smaller stem‐loop (Supporting Figure 12). When adding only Gdm^+^ in the absence of Mg^2+^, no ligand binding was observed (Supporting Figure S4). Specific Mg^2+^ binding to P1 is required for Gdm^+^ binding, and pre‐formation of the kissing loop‐loop conformation precedes ligand binding. The Mg^2+^‐bound state coexists with both, the Mg^2+^‐free state and the Mg^2+^/Gdm^+^‐bound state.

From quantification of CSPs in ^1^H‐1D NMR titration experiments (Supporting Figure S5), we determined a K_D_ of 130 μM for the full‐length aptamer Gdn49 wt with a Hill coefficient n of 1.1 and a K_D_ of 200 μM with n of 1.2 for the isolated P1 stem‐loop Gdn23 wt (Figure 3D and 3E). While the Gdn49 wt might be expected to bind two ligand molecules the Hill coefficient points to a strong difference in affinity between the two ligand binding stems P1 and P2. Gdn23 wt features an *ACGA* loop motif and the K_D_ values of Gdn23 wt and Gdn49 wt are in a comparable regime (Table 2 and Figure 3E). Accordingly, in Gdn49 wt the *ACGA* loop motif located in P1 represents the stronger ligand binding site. Since the K_D_ value for the isolated stem‐loop is higher, we conclude that the kissing hairpin formation upon ligand binding is facilitated by linker L1/2 and the additional interaction with stem‐loop P2.

### DOSY and SAXS characterization

Characterization of ligand binding by ^1^H‐1D NMR revealed two new G‐imino signals, assigned to the formation of two additional GC base pairs. These base pairs may either arise from intramolecular kissing loop interaction or from dimerization of Gdn49 wt via intermolecular P1‐P1’ kissing loop interaction. The lack of any CSPs after addition of ligand, clearly pointed to the induction of dimerization as the more likely event. Thus, to determine the molecularity of the ligand‐Gdn49 wt complex, we measured its hydrodynamic radius (R_h_) by diffusion ordered spectroscopy (DOSY) NMR experiments.^[28]^ We observed an increase of R_h_ greater than 60 % upon addition of Gdm^+^, indicating dimerization upon complex formation (Supporting Figure S6). In conjunction with the ^1^H‐1D‐NMR data showing a single set of signals, we assume the formation of a C_2_‐symmetric dimer with the P1 loop as interaction interface (Supporting Figure S7). The addition of only Mg^2+^ increased R_h_ by 10 % (Supporting Figure S6), pointing to a Mg^2+^‐dependent transient pre‐formation of kissing loop interaction.

**Figure 4:**
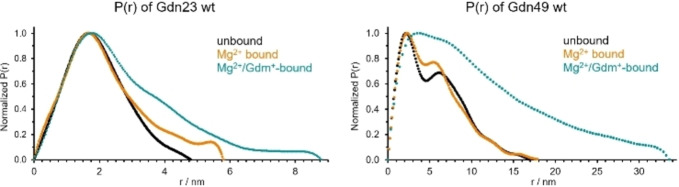
SAXS analysis of guanidine‐II for Gdn23 wt (left) and Gdn49 wt (right). These curves represent the distance distribution functions P(r) for unbound (black), Mg^2+^ bound (orange) and Mg^2+^/Gdm^+^‐bound (cyan) RNA reporting D_max_ as measurable geometric parameter.

In addition, we characterized Gdn23 wt and Gdn49 wt by SAXS focusing on the oligomerization states of the free and complexed RNAs (Figure 4, Supporting Figure S8 and Supporting Table S9), as previously conducted for different riboswitches.[^29^, ^30^] For Gdn23 wt in the absence of Mg^2+^ and Gdm^+^, analysis of SAXS data revealed an apparent molecular weight (MW) of 7.3 kDa, a maximal intramolecular distance (D_max_) of 48 Å and a radius of gyration (R_g_) of 14.8 Å consistent with a predominantly monomeric conformation (Table 1). These data also agree with a predicted structure^[31]^ as well as the crystal structure^[19]^ (Supporting Figure S10). Upon addition of Mg^2+^, we also observed an increase of the apparent MW (16 %), D_max_ (21 %) and R_g_ (20 %). When Gdm^+^ is added, the apparent MW increases to 14.2 kDa, D_max_ to 88 Å and R_g_ to 22 Å in accordance with a dimeric species, which is in line with the NMR data. The theoretical molecular weight of the full aptamer domain (Gdn49 wt) is 14.9 kDa, but analysis of SAXS data for free Gdn49 wt revealed an apparent molecular weight of 30.0 kDa in line with predominantly dimeric arrangement. Since the P1‐P1’ dimerization is not observed in the free Gdn23 wt containing only the P1 stem, we conclude that there is at least a weak interaction of the P2 stems, which, however, is ligand‐independent. We speculate that this potential P2‐P2’‐based dimerization stems from the interaction of G‐rich P2 hairpins. The P2 hairpins might interact via G‐mediated interactions in line with the weak NMR signals presumably due to G−G Hoogsteen interaction observed in the corresponding region (10.3 ppm, Supporting Figure S2). Addition of only Mg^2+^ to Gdn49 wt led to an increase in molecular weight (18 %) consistent with the increase in R_h_ observed in the DOSY NMR experiments (see also R_g_/R_h_ in Supporting Table S11). When both, Mg^2+^ and Gdm^+^ were added, SAXS data yielded an experimental molecular weight of at least 94 kDa. This high molecular weight indicates conformations of higher molecularity, presumably hexamers, most likely stabilized by intermolecular P1‐P1’ interactions.

**Table 1:** SAXS‐derived geometric parameters for guanidine‐II aptamer constructs Gdn23 wt and Gdn49 wt. We calculated the expected values using monomer and dimer models,^[31]^ respectively.

SAXS analysis		Unbound	Mg^2+^‐bound	Mg^2+^/Gdm^+^‐bound	Expected changes from unbound to bound states
Gdn23 wt	MW_exp_ [kDa]	7.3	8.5	14.2	7.0 to 14.0
	D_max_ [Å]	48	58	88	42 to >80
	R_g_ [Å]	14.8	17.8	22.0	13.6 to 21.4
	Conformation	monomer	>monomer	dimer	monomer to dimer
					
Gdn49 wt	MW_exp_ [kDa]	30.0	35.5	>94.0	14.9 to 14.9 (for intramolecular interaction) or to 29.8 (for intermolecular interaction)
	D_max_ [Å]	169	181	>336	no realistic determination possible due to linker
	R_g_ [Å]	40.7	42.0	83.3	no realistic determination possible due to linker
	Conformation	dimer	>dimer	>hexamer?	monomer to kissing loop‐loop interaction

### Investigation of mutants

**Figure 5:**
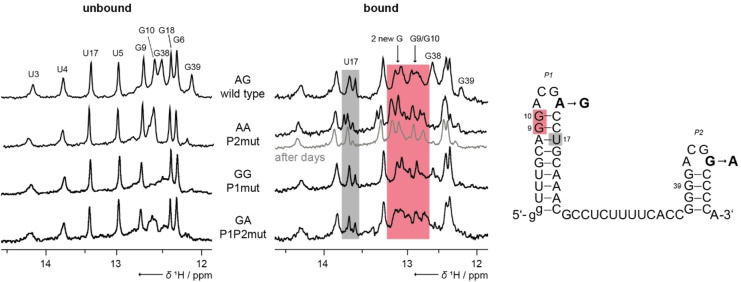
Investigation of mutated constructs shows distinct CSPs in P2. ^1^H‐1D NMR spectra of ligand titration experiments for Gdn49 constructs of wild‐type and mutants are shown: free forms are shown on the left, and in presence of 5 mM Mg^2+^ and 1 mM Gdm^+^ on the right. CSPs marked in gray for U17 and in red for G9, G10 and two new arising signals, respectively. Data were measured at 600 MHz, room temperature, 4096 points, 512 scans with 20 μM of RNAs. An overview of the constructs and mutations is given in Table 2.

Due to the lack of experimental data for the G variant of the fourth loop nucleotide (G44) we investigated whether G‐to‐A mutants in P2 could promote P1‐P2 interactions. Since Gdn13 wt features a *ACG**G*** motif, we first examined the G44A mutant, both in the isolated stem‐loop (Gdn13 G44A, Supporting Figure S12) and in the full‐length aptamer (Gdn49 P2mut, Figure 5). This mutation led to ligand binding, as we observed significant CSPs for both constructs (Gdn13 G44A and Gdn49 P2mut) upon Gdm^+^ titration (Supporting Figure S13). We conclude that the loop sequence *ACGR* with an A at loop position 4 enables ligand binding. P1 dimerizes via P1‐P1’ kissing loop interaction, if P2 contains a *ACGG* loop motif with impaired kissing loop‐loop interaction potential. Accordingly, we further speculated that an intramolecular kissing loop‐loop complex could be formed in the presence of two ACGA loop motifs. Therefore, we investigated all four combinations of A/G loop variations (Table 2) in ligand titrations (Figure 5 and Supporting Figure S13) and determined K_D_ values (Supporting Figure S14).

**Table 2:** Loop variants of the investigated guanidine‐II aptamer construct and its binding behavior at RNA concentrations of 50 μM with 5 mM Mg^2+^ and up to 8 mM Gdm^+^.

	Loop sequence	Abbreviation for loop sequence	K_D_ [μM]	Hill coefficient n
P1 wild‐type Gdn23	ACGA	A	197.4±8.8	1.16±0.06
P2 wild‐type Gdn13	ACGG	G	not detected	not detected
Aptamer wild‐type Gdn49	ACGA…ACGG	AG	133.4±3.9	1.10±0.04
Gdn49 P1mut	ACGG…ACGG	GG	264.3±27.8	1.35±0.15
Gdn49 P2mut	ACGA…ACGA	AA	58.9±8.1	1.01±0.22
Gdn49 P1P2mut	ACGG…ACGA	GA	165.0±14.3	1.38±0.17

In NMR Gdm^+^‐titrations with all possible loop combinations – AG (wild‐type), AA, GG, GA – we observed approximately the same CSP response – U17, G9, G10 show CSPs, and two new G‐signals arise. Consequently, mutants with *ACG**G*** loop motifs are ligand‐binding competent and the ligand is clearly sensed by both types of loop motifs in the context of P1. Comparison of K_D_s between constructs (Table 2) shows a clear preference for A‐loop interactions, evidenced by the lowest K_D_ of 60 μM for the AA‐construct (Gdn49 P2mut), and followed by the construct with an A‐loop in only P1 (AG, Gdn49 wt, 130 μM). Accordingly, the K_D_ value is higher for mutants with either an A‐loop only in P2 (165 μM, GA, Gdn49 P1P2mut), and yet more when only the G‐loop is present (260 μM, GG, Gdn49 P1mut). Our NMR and SAXS data showed that only P1 – the A‐loop construct – adopts a monomeric conformation and the aptamer wild‐type construct (Gdn49 wt) has a dimer conformation in the free form. Comparing the K_D_ values relative to wild‐type, it becomes apparent that ligand binding and formation of binding pocket are compromised either by shortening the construct to only P1 or when mutated to the G variant. In contrast, a mutation to the A‐loop motif improves binding by a factor of 2.

We thus propose that the binding pocket is preferentially formed when adenosines are presented in position four of the loop and a P2‐P2’ interaction supports dimerization because the stem is GC rich, and especially when it contains the G‐loop motif. Possibly, the enthalpic gain from interactions involving G‐loops might not sufficiently compensate the entropic penalty compared to sequences involving interactions only between A‐loops. Consequently, a G‐loop only mutant is less capable of forming binding pockets. We speculate that this is due to a possible weak intramolecular loop base pair interaction between C12‐G14 and C42‐G44, respectively. This finding implies that a single nucleotide can be responsible for the binding efficiency of the entire riboswitch as one ligand seems to be sufficient to promote the kissing interaction. In fact, a G variation in both loop sequences is rarely found in nature and the *ACG**A*** variant of P1 is conserved in most cases.^[23]^ Moreover, for the AA‐construct (P2mut), we observed that after a few days the bound state shows the exact same CSP scheme as the wild‐type (Figure 5, spectra highlighted in gray). We thus conclude, the kissing loop‐loop interaction induces an intermolecular C_2_‐symmetric dimerization rather than an intramolecular interaction. Such change of conformation takes also place in the unbound states of both, the Gdn13 G44A‐mutant and the corresponding aptamer Gdn49 P2mut, which within a few days show the same set of signals as the wild‐type constructs (Supporting Figure S15).

## Conclusion

In this study, we probed several wild‐type and mutated constructs of the guanidine‐II riboswitch from *E. coli* with a particular focus on ligand binding capacity and molecularity in an *in vitro* context. Assessing the interaction with Gdm^+^, we identified a striking difference between *ACGA* and *ACGG* loop motifs. While *ACGA* motifs tend to show kissing loop interactions together with Gdm^+^ as postulated in earlier studies, *ACGG* motifs interact prior to Gdm^+^ addition rendering them unavailable for intramolecular action. This is supported by SAXS and DOSY NMR data which indicate higher‐order oligomers of Gdn49 wt under Gdm^+^ influence. In mutational studies, we observed that P1 and P2 are involved in (intramolecular) ligand recognition if adenosines are present in position four in the loop. If a single G variant is present, an intermolecular A‐loop to A‐loop interaction is preferred. In GG‐constructs, the ligand is recognized but we detect a lower affinity. In addition, AG‐constructs are likely influenced by P2‐P2’ interaction, responsible for wild‐type oligomerization and therefore might bind ligand intermolecularly. In a biological context, P2‐P2’ interactions via G‐mediated contacts are unlikely to occur but remain important to bear in mind for chemical biology applications. The reduced activity of riboswitch variants bearing an *ACGG* loop motif however can be considered a highly abundant tuning mechanism towards the extent of translational control. This is in line with the low abundance of GG variants in biological systems.

## Experimental Section

### Constructs of interest

The sequence of the *Escherichia coli* SugE guanidine‐II riboswitch aptamer (Gdn‐II, mini‐ykkC RNA motif) was used (Figure 2B).

The construct is a composite of P1 (23 nt) and P2 (13 nt) hairpins and the native linker L1/2 (13 nt) connecting both. The 5’ end was modified with G1 and G2 to enable an efficient *in vitro* transcription^[32]^ and stabilize the P1 hairpin.

The mutant P1mut was modified with G instead of A14 and P2mut with A instead of G44.

### RNA preparation

The wild‐type RNA constructs were purchased from Dharmacon Inc.

In addition, Gdn23 wt, Gdn49 wt and all its mutants were prepared in house through *in vitro* transcription with T7 RNAP.^[33]^

DNA templates included the necessary T7 promoter sequence and were either obtained from linearizing plasmid from pUC57 vector (GenScript) containing the full‐length native sequence of Gdn‐II riboswitch or PCR run‐off according to the standard protocol by New England Biolabs® (0.5 mM of each primer, 200 mM dNTPs) using homemade Phusion polymerase. The primers were purchased from Eurofins Genomics (Germany).

Transcription reactions contained transcription buffer (100 mM Tris/glutamate pH 8.1), 2 mM spermidine, 20 mM dithiothreitol (DTT), 20 % (v/v) DMSO, 5 mM of each NTP, 10 mM Mg(OAc)_2_, 9.6 μg/mL of homemade yeast inorganic pyrophosphatase (YIPP) and 32 μg/mL of homemade T7 RNAP. Unlabeled NTPs were purchased from Carl Roth GmbH+Co. KG (Germany) and ^13^C,^15^N labeled NTPs from Silantes GmbH (Germany) or from Eurisotop (France).

The conditions were optimized for yield and sample purity. The *in vitro* transcription was performed in 15 mL scale. Purification was performed either by HPLC, preparative PAGE or buffer exchange to NMR buffer if necessary.^[34]^

### NMR spectroscopy

NMR samples were prepared by adding 8 % D_2_O and 7.5 nmol DSS as internal reference to RNA stock solutions in NMR buffer (25 mM potassium phosphate buffer, 50 mM KCl, pH 6.2). All spectra were recorded of 280 μL samples in Shigemi NMR tubes (Shigemi Inc.)

NMR experiments were conducted on Bruker AV600, AV700, AV800, AV900 and AV950 spectrometers, equipped with cryogenic probes. Data were processed with Bruker TopSpin® 3.6.1 (Bruker Biospin) and NMRFAM‐SPARKY 3.135.^[35]^ Water suppression was achieved using WATERGATE^[36]^ or jump‐and‐return echo^[37]^ water suppression pulse schemes.

Analysis of dissociation constants (K_D_) were carried out by measuring intensities of the imino proton signals of U17 in ^1^H‐1D spectra for Gdm^+^ binding to the Mg^2+^‐RNA‐complex using the one‐site binding hyperbole (equation 1) or Hill formalism (equation 2) as fitting function.[^17^, ^38^] In equation 2, the postulated cooperativity n is considered. The unbound signal was correlated with the bound signal, then normalized and subsequent fitted while leaving the RNA concentration variable.

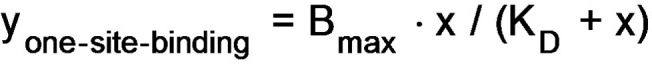




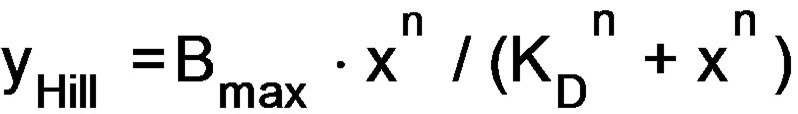




DOSY measurements were evaluated either by using the mean values of the aromatic range or all ranges to obtain the logarithmic value for the diffusion constant D (Supporting Figure S6). Compared to the internal standard 1,4‐dioxane with known hydrodynamic radius of 2.14 Å, the hydrodynamic radius of the investigated Gdn49 wt is determined via the equation 3:

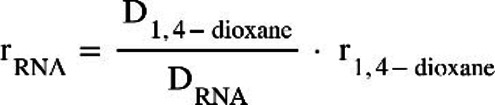




### SAXS

SAXS measurements were performed on BM29 BioSAXS beamline of the European Synchrotron Radiation Facility (ESRF) in Grenoble, France in a remote measurement mode. Sample buffers were identical to NMR conditions except for D_2_O. Sample volumes were 60 μL and the following concentrations have been used 2.5 mg/mL for free and Mg^2+^‐bound and 2 mg/mL for Mg^2+^/Gdm^+^‐bound guanidine‐II aptamer wild‐type constructs (Gdn23 wt and Gdn49 wt). We also measured all samples at higher concentrations of 4–5 mg/mL. We consistently used the more dilute sample for downstream analysis in order to avoid non‐specific effects from concentration with respect to monomer‐dimer‐oligomer equilibria. Notably, also at lower concentrations, both the S/N and data quality were of sufficient quality. We used the respective filtrates during sample concentrating as buffer match to subtract background scattering. Samples were measured by exposure at 12.5 keV beam energy, scattering was acquired on a Pilatus3 2 M detector placed under vacuum, and with local standard setups of distance and beam size. The herein used q‐range for RNA samples was 0.25–5.2 nm^−1^. Acquired frames were automatically scanned for sufficient quality and to exclude radiation damage and summed up over a period of 5–10 sec of measurement. Buffer subtraction was performed using an average from prior and posterior buffer‐only runs. For data analysis, both the pre‐processed data were visualized manually and the estimation of R_g_, D_max_ and MW was carried out using ATSAS 3.0.^[39]^ Structural alignment was performed using the SASpy PyMOL plugin.^[40]^

## Conflict of interest

The authors declare no conflict of interest.

1

## Supporting information

As a service to our authors and readers, this journal provides supporting information supplied by the authors. Such materials are peer reviewed and may be re‐organized for online delivery, but are not copy‐edited or typeset. Technical support issues arising from supporting information (other than missing files) should be addressed to the authors.

Supporting Information

## Data Availability

The data that support the findings of this study are available from the corresponding author upon request.
