# Supplementary material for: Characterization of Structure and Dynamics of the Guanidine‐II Riboswitch from Escherichia coli by NMR Spectroscopy and Small‐Angle X‐ray Scattering (SAXS)
Source: Chembiochem. 2021 Dec 9;23(3):e202100564. doi: 10.1002/cbic.202100564 (PMC9300104; doi:10.1002/cbic.202100564)
Supplement: Supplementary file 1 — Supporting Information [file CBIC-23-0-s001.pdf]

ChemBioChem

Supporting Information

Characterization of Structure and Dynamics of the Guanidine-II Riboswitch from *Escherichia coli* by NMR Spectroscopy and Small-Angle X-ray Scattering (SAXS)

Tatjana Schamber, Oliver Binas, Andreas Schlundt, Anna Wacker, and Harald Schwalbe*

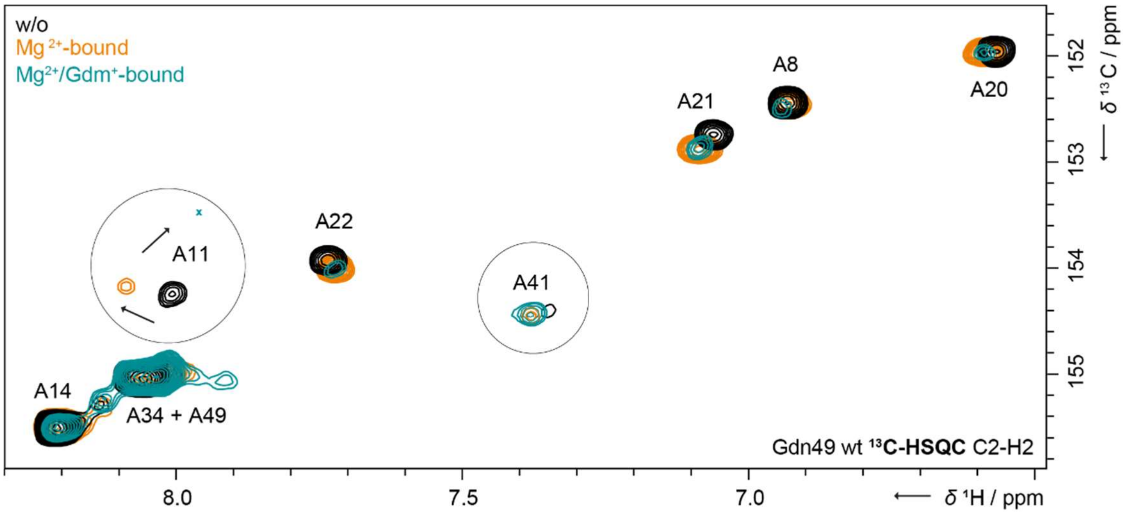

Supplementary Figure S1: ^{13}C-HSQC spectra of adenosine C2-H2 recorded from Gdn49 wt for unbound, in presence of 5 mM Mg^{2+} and in addition of 1 mM Gdm^{+}. Indicate only CSP for Mg^{2+} interaction, but no additional shift for Gdm^{+} interaction in P2, to see at A41. This contrasts with the comparable loop nucleotide from P1 (A11). This signal becomes larger with increasing Gdm^{+}-concentration, while the signals for the stem adenosines of P1 decrease even further. Data were measured at 800/201 MHz, 1024 x 128 points, 16 scans with 500 µM ^{13}C,^{15}N-labeled Gdn49 wt.

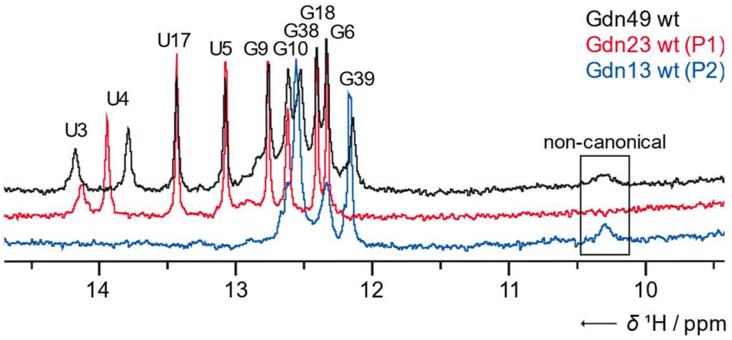

Supplementary Figure S2: Weak NMR signals at 10.3 ppm are indicating a non-canonical base pair. There are the imino regions of ^{1}H-1D NMR spectra for unbound constructs of Gdn49 wt (black), Gdn23 wt (red) and Gdn13 wt (blue) shown. Data were measured at 600 MHz, room temperature, 4096 points and 1024 scans. Each sample contained 20 µM of RNA.

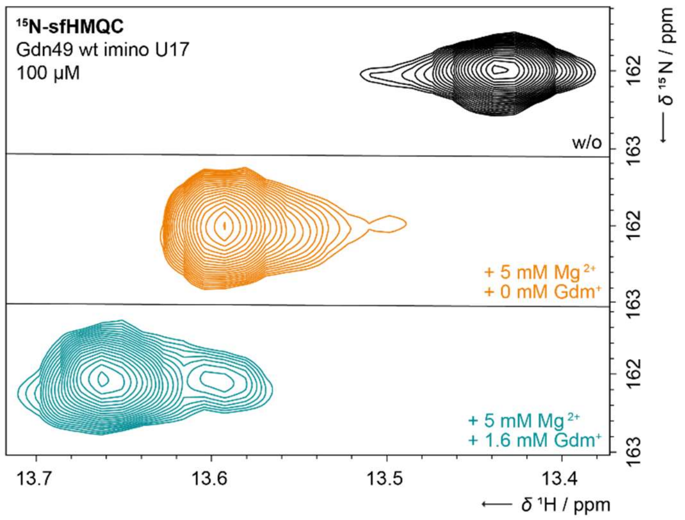

Supplementary Figure S3: Imino signal of U17 in 2D-SOFAST-HMQC spectra for ^{1}H-1D NMR spectra of Gdn49 wt unbound, in presence of 5 mM Mg^{2+} and in addition of 1.6 mM Gdm^{+}. In the presented Mg^{2+}/Gdm^{+}-bound state (cyan), two coexisting states are still present. Data were measured at 600 MHz, room temperature, 36 points and 64 scans. Sample contained 100 µM RNA of Gdn49 wt.

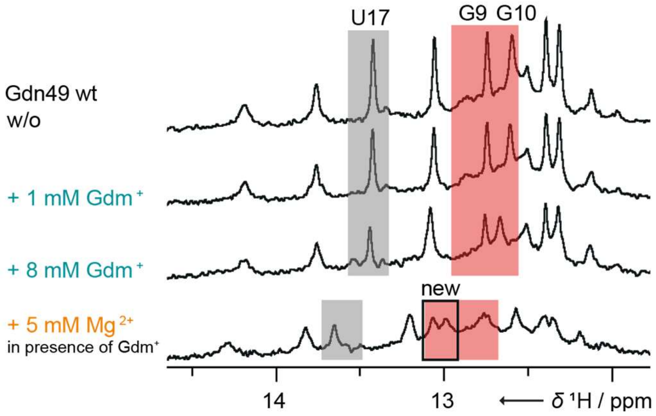

Supplementary Figure S4: Gdm^+ addition initially without Mg^{2+}. ^{1}H-1D NMR spectra of Gdn49 wt unbound, in presence of 1 mM or 8 mM Gdm^+ and in addition of 5 mM Mg^{2+}. Data were measured at 600 MHz, room temperature, 4096 points and 128 scans. Sample contained 100 µM RNA of Gdn49 wt.

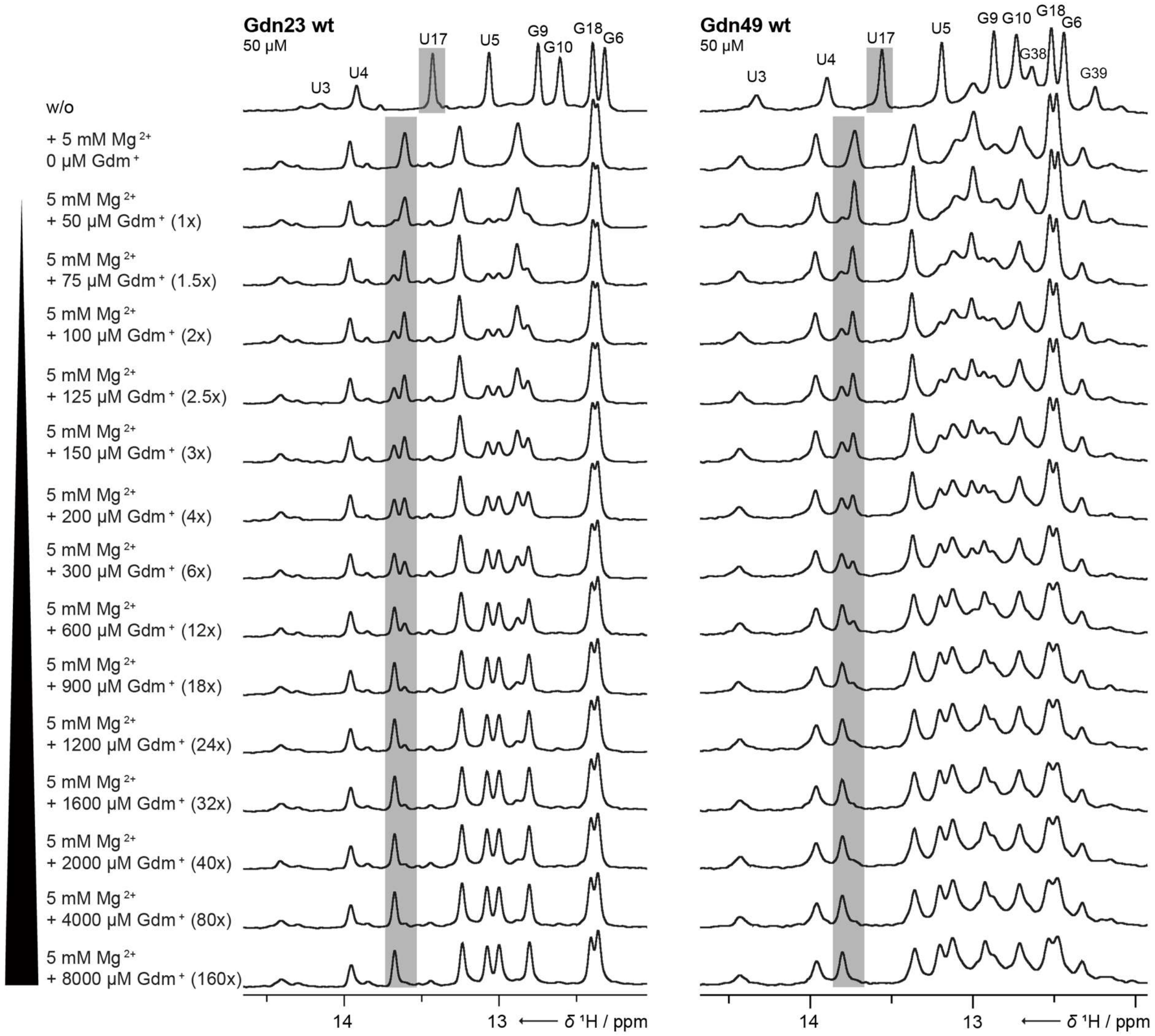

Supplementary Figure S5: Ligand titration ^{1}H-1D NMR spectra of Gdn23 wt and Gdn49 wt from unbound, in presence of 5 mM Mg^{2+} and up to 8 mM Gdm^+. Signal intensities of de- and increasing imino signal of U17 (marked in gray) were used for K_D determinations. Data were measured at 700 MHz, room temperature, 2048 points and 768 scans. Sample contained 50 µM RNA.

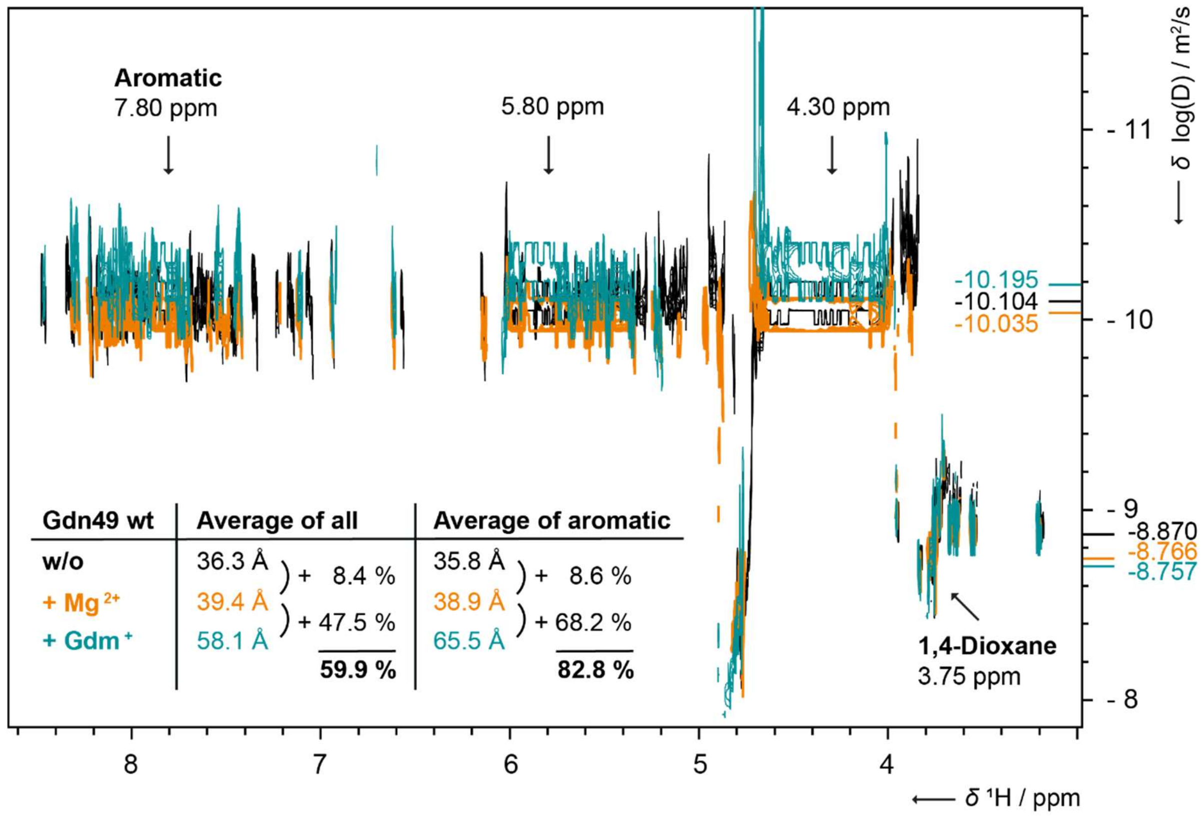

Supplementary Figure S6: Determination of hydrodynamic radii in pseudo-2D DOSY spectra
Pseudo-2D diffusion ordered spectroscopy (DOSY) NMR spectra of Gdn49 wt unbound (black), in presence of 5 mM Mg^{2+} (orange) and in addition of 1 mM Gdm^{+} (cyan). Data were measured at 900 MHz, room temperature, 16384/64 points, 112 scans, 120 µs diffusion pulse gradient length and 4.5 ms diffusion time. Sample contained 100 µM RNA of Gdn49 wt.
The labeled ranges at 4.30 ppm, 5.80 ppm and 7.80 ppm as well as the internal standard 1.4-dioxane at 3.75 ppm were used for analyzing. The average values for log(D) of all and of 1,4-dioxane are annotated, using equation 1, the reported values are obtained.

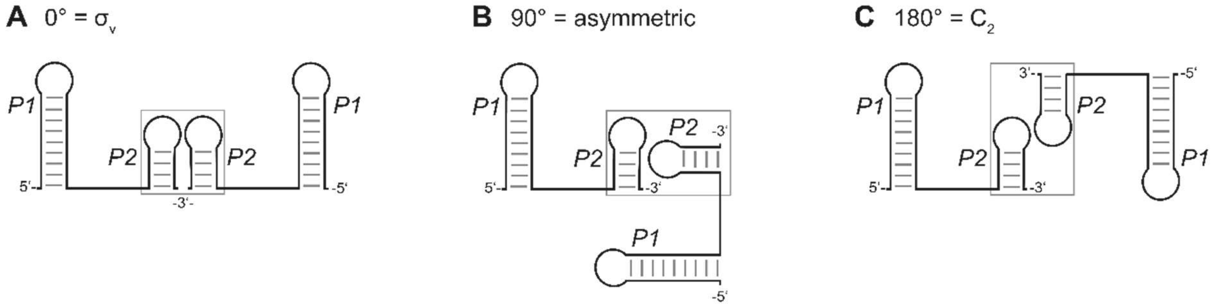

Supplementary Figure S7: Models of intermolecular dimer for Gdn49 wt.
The orientation of the two molecules is shown schematically from 0°, 90° and 180° to each other. For only possibility of the angle 180° (C) could result in only one set of NMR signals as obtained in the experiments. For this reason, we assume a C_2 symmetric dimerization.

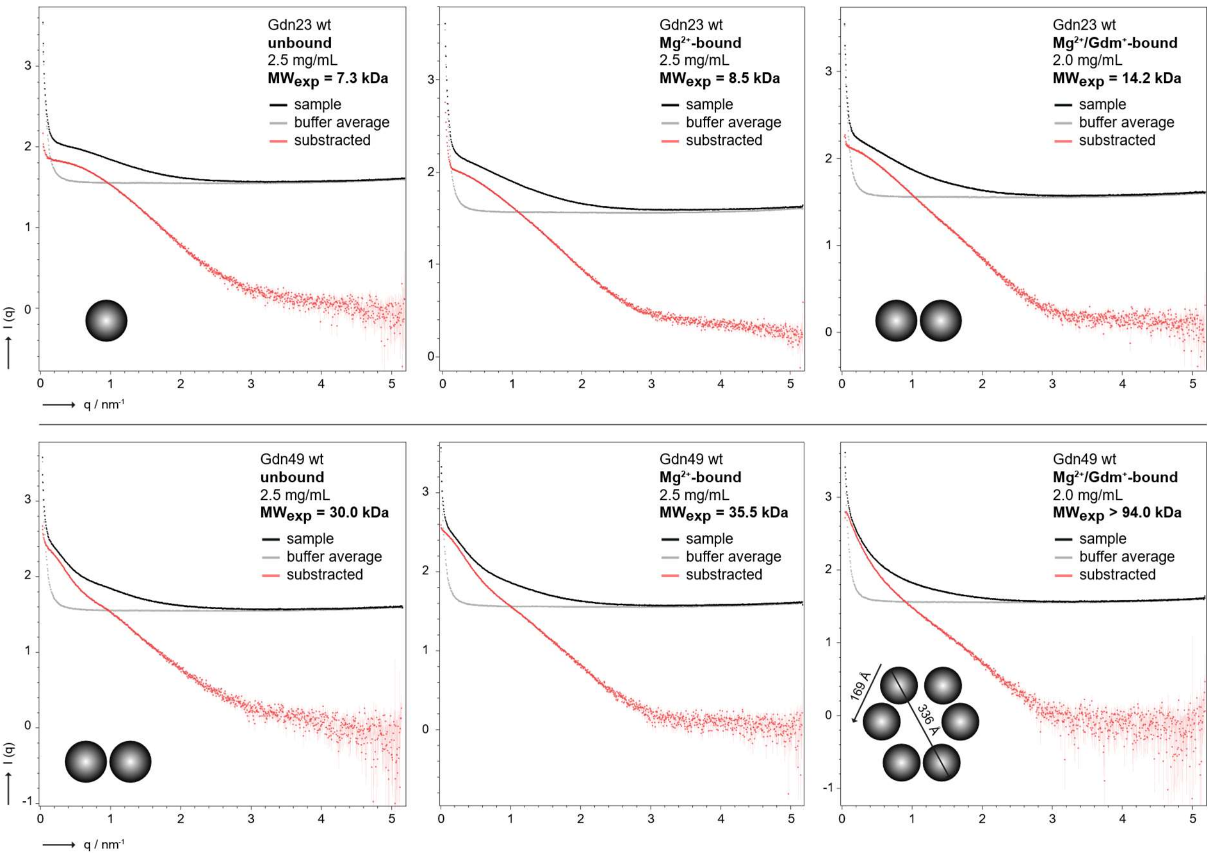

Supplementary Figure S8: SAXS scattering curves of Gdn23 wt (top row) and Gdn49 wt (bottom row) for unbound (left), Mg^{2+}-bound (middle) and Mg^{2+}/Gdm^{+}-bound (right) states, respectively.

These curves represent the scattering profiles for buffer average (gray), sample (black) and buffer-subtracted samples (red). The most likely oligomeric states and their arrangements based on D_{max} and R_g are illustrated in schematic representations via balls.

Supplementary Table S9: Usage of SAXS scattering curve raw data or fitting and deriving of D_{max} and R_g from Gdn23 wt and Gdn49 wt by using ATSAS 3.0[39].

**Gdn23 wt**	**unbound**	**Mg^{2+}-bound**	**Mg^{2+}/Gdm^{+}-bound**
q-range used for data fit [nm^{-1}]	0.25-4.82	0.33-4.50	0.26-3.57
q-range used for data fit [points]	42-930	58-868	44-686
Total quality estimate resulted for P(r) curve	0.81	0.54	0.63
**Gdn49 wt**	**unbound**	**Mg^{2+}-bound**	**Mg^{2+}/Gdm^{+}-bound**
q-range used for data fit [nm^{-1}]	0.18-1.96	0.17-1.64	0.07-1.28
q-range used for data fit [points]	29-374	27-360	7-242
q-range used for data fit [points]	0.66	0.70	0.61

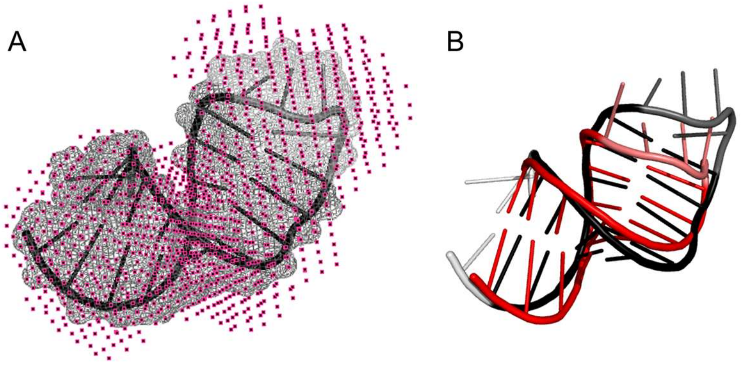

Supplementary Figure S10: Visualization of unbound Gdn23 wt

(A) Superimposition of the predicted structure[31] of unbound Gdn23 wt (black, *ACGA* loop highlighted in gray) with a de-novo built dummy model (shown as dots)[40] based on experimental SAXS data. The normalized spatial discrepancy (NSD) is 1.82.

(B) Comparison of the predicted structure[31] in A (black, *ACGA* loop highlighted in gray) with the crystal structure from *E. coli* (PDB: 5NDI)[19] (red, *ACGA* loop highlighted in light red). The predicted structure as well as investigated Gdn23 wt differ in the nucleobases G1, G2 and C23 (highlighted in white) to the crystal structure (20 nt). The NSD is 1.65.

Supplementary Table S11: Comparison of radius of gyration (R_g) obtained with SAXS and hydrodynamic radius (R_h) from DOSY NMR measurements.
The two similar parameters R_g and R_h provide an approximate measure or the biologically relevant structure of free RNA and its ligand-binding complex in solution. The closer the actual structure resembles a sphere, the more likely R_g and R_h tend to be equal. The characteristic shape factor of a sphere R_g/R_h is 0.775 which means that R_g is smaller than R_h. Higher values of R_g/R_h as for Gdn49 wt mean that the molecules differ from globular to non-spherical or elongated structures and R_g becomes larger than R_h, respectively.[41]

**Gdn49 wt**	**unbound**	**Mg^{2+}-bound**	**Mg^{2+}/Gdm^{+}-bound**
R_g	40.7	42.0	83.3
Average value of R_h	36.1	39.2	61.8
**R_g/R_h**	1.13	1.07	1.35

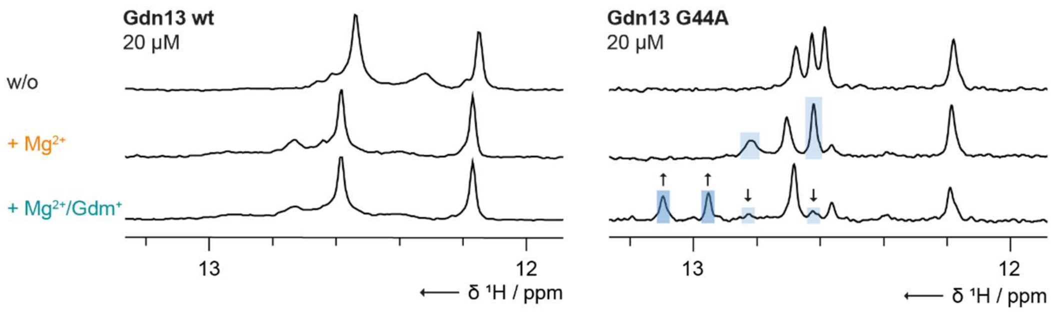

Supplementary Figure S12: Ligand binding capacity for P2

There are shown the imino regions of ^{1}H-1D NMR spectra of Gdn13 wt (left) and Gdn13 G44A mutant (right) unbound, in presence of 5 mM Mg^{2+} and in addition of 1 mM Gdm^{+}. There are no de- and increasing signals for wild-type construct, but in case of G44A mutant, they are highlighted and marked with arrows. Data were measured at 600 MHz, room temperature, 4096 points and 1024 scans. Sample contained 20 µM RNA.

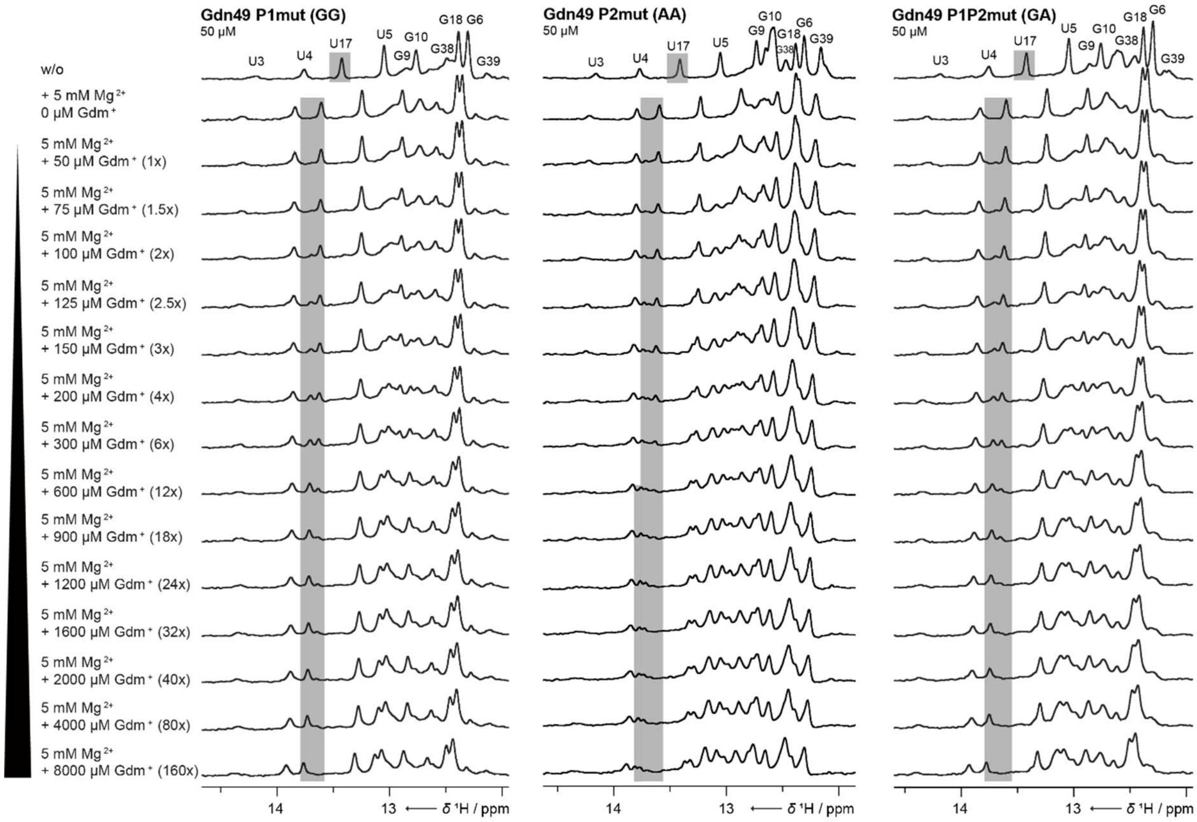

Supplementary Figure S13: Ligand titration ^{1}H-1D NMR spectra of Gdn49 mutants from unbound, in presence of 5 mM Mg^{2+} and up to 8 mM Gdm^{+}. Signal intensities of de- and increasing imino signal of U17 (marked in gray) were used for K_D determinations. In case of Gdn49 P2mut (AA), the two increasing signals (instead of only one) were summed for determination of the K_D. Data were measured at 800 MHz, room temperature, 2048 points and 768 scans. Samples contained 50 μM RNA.

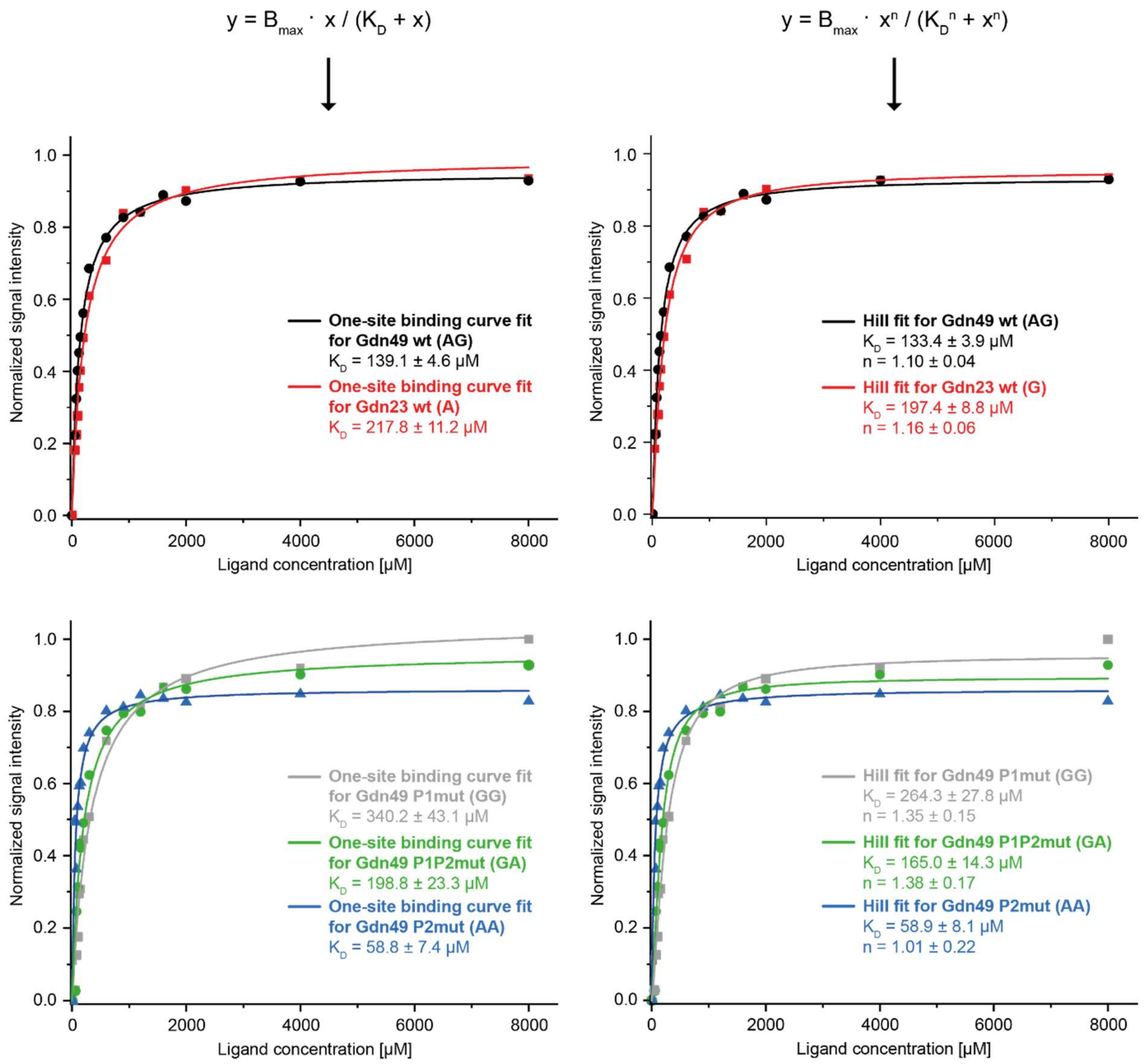

Supplementary Figure S14: Plot of K_D-determination via ^{1}H-1D NMR titration experiments for imino signal U17 of investigated wild-type and mutant constructs: Gdn49 wt (AG) in black, Gdn23 wt (G) in red, P1mut (GG) in gray, P1P2mut (GA) in green and P2mut (AA) in blue. The resulted values were determined by fitting the one-site binding curve as hyperbolic function (on the left) and the Hill equation considering cooperativity (on the right).

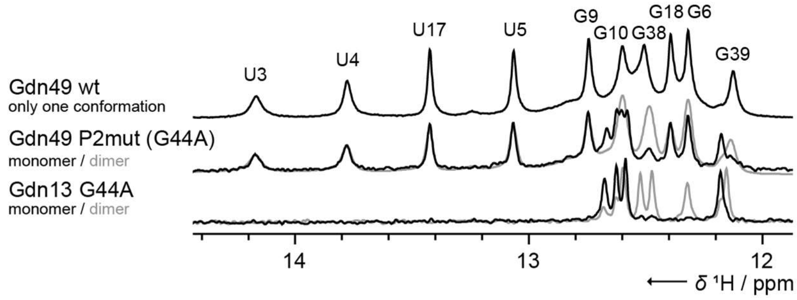

Supplementary Figure S15: Conformational transformation in unbound state of G44A mutants

There are shown the imino regions of ^{1}H-1D NMR spectra of unbound Gdn49 wt, Gdn49 P2mut and Gdn13 G44A mutant. Illustrated in gray are the spectra of mutants measured a few days later or higher concentrations, respectively.
